# WWP1 modulates metabolic adaptation in white adipose tissue but does not significantly modify caloric restriction-induced longevity in mice

**DOI:** 10.3389/fnut.2026.1846488

**Published:** 2026-06-30

**Authors:** Yuka Nozaki, Kumi Miura, Kenta Yajima, Shunsuke Hoshino, Shoma Tamori, Yuhei Mizunoe, Masaki Kobayashi, Yoshikazu Higami

**Affiliations:** 1 Faculty of Pharmaceutical Sciences, Tokyo University of Science, Tokyo, Japan; 2 Division of Aging Biology, Research Institute for Science and Technology (RIST), Tokyo University of Science, Tokyo, Japan; 3 Department of Nutrition and Food Science, Graduate School of Humanities and Sciences, Ochanomizu University, Tokyo, Japan; 4 Institute for Human Life Science, Ochanomizu University, Tokyo, Japan; 5 Division of Cell Fate Regulation, Research Institute for Biomedical Science (RIST), Tokyo University of Science, Chiba, Japan

**Keywords:** caloric restriction, *de novo* fatty acid synthesis, longevity, mice, WWP1

## Abstract

Caloric restriction (CR) extends lifespan across diverse species. While the WW domain-containing E3 ubiquitin ligase1 (WWP1) is an essential mediator of dietary restriction-induced longevity in *Caenorhabditis elegans*, its role in mammalian CR remains unclear. In this study, we investigated the role of WWP1 in CR-mediated longevity and metabolic adaptation using systemic *Wwp1* knockout (KO) mice. Male wild-type (WT) and *Wwp1* KO mice were subjected to either *ad libitum* (AL) feeding or long-term CR (70% of AL intake) and monitored throughout their natural lifespan. As a result, *Wwp1* deficiency did not markedly modify the CR-associated survival response in mice. Despite comparable food intake, CR-*Wwp1* KO mice after middle age exhibited a modest increase in body weight compared with CR WT mice. WWP1 deficiency selectively enhanced CR-induced, Srebp-1c-dependent expression of proteins involved in *de novo* fatty acid synthesis in epididymal WAT, whereas hepatic lipid metabolism was unaffected. Collectively, our results demonstrated that, unlike in *C. elegans*, WWP1 is not required for CR-induced longevity in mammals; rather, it acts as a partial suppressor of CR-driven *de novo* fatty acid metabolism, limits energy storage and lipid retention in WAT.

## 1 Introduction

Caloric restriction (CR), also known as dietary restriction (DR), is a well-established and reproducible intervention that delays the onset of many age-related pathophysiological changes and extends lifespan across diverse species, including yeast, worms, and mammals (1). The beneficial effects of CR have been associated with reduced GH/IGF-1 and mTOR signaling, increased sirtuin activity and mitochondrial biogenesis, and decreased oxidative stress and inflammation; however, the underlying mechanisms remain incompletely understood (2).

WW domain-containing E3 ubiquitin ligase1 (WWP1)–also known as TGIF-interacting ubiquitin ligase 1 (TIUL1) or atrophin-1 interacting proteins (AIP5) in mammals–is a homologous to E6AP C-terminus (HECT)-type E3 ubiquitin protein ligase (3). WWP1 belongs to the NEDD4 family, which includes NEDD4-1, NEDD4-2, WWP2, itchy E3 ubiquitin protein ligase (ITCH), NEDD4-like E3 ubiquitin protein ligases (NEDL 1 and 2), and SMAD-specific E3 ubiquitin protein ligases (SMURF 1 and 2) (4–6). WWP1 contains a C2 domain at its N-terminus, four WW domains in its central region, and a HECT domain at its C-terminus (7). It is ubiquitously expressed in most human tissues and has been implicated in a broad spectrum of pathologies, including various cancers, viral infections, muscular dystrophy, neurological disorders, and aging. These observations underscore the role of WWP1 as a key regulator of diverse intracellular processes (8).

We previously suggested that WWP1 functions as a metabolism-related factor, particularly in white adipose tissue (WAT) and the liver, in an obese model. In WAT, high-fat diet-induced obesity increases WWP1 expression, which in turn suppresses *β*-3 adrenergic receptor expression and lipolysis (9, 10). In contrast, in the liver, WWP1 increases PTEN expression and suppresses insulin signaling, thereby exacerbating fat accumulation (11). These results suggest that WWP1 acts as an exacerbating factor in obesity-related lipid metabolic dysfunction in the WAT and liver of obese mice (12).

Previously, Carrano and colleagues demonstrated that the WWP1 ubiquitin pathway is essential for DR-induced longevity in *Caenorhabditis elegans* (13–15). In 2009, they showed that increased *Wwp1* expression (GFP: WWP-1) extended lifespan (by up to 20%), even under *ad libitum* feeding conditions. Consistent with this finding, reduction of *Wwp1* completely suppressed DR-induced lifespan extension, and a catalytically inactive mutant (dominant-negative WWP-1 [C762A]) also inhibited DR-induced longevity (13). In 2014, to identify the WWP1 substrates, the authors performed a targeted RNAi screen and identified the Krüppel-like transcription factor KLF-1 as a key regulator of DR-induced longevity. Furthermore, they showed that the intestine is a major site of WWP1/KLF-1 action, and that intestinal *Klf-1* knockdown suppresses the longevity of WWP1-overexpressing worms (14). Collectively, these studies indicate that WWP1-mediated ubiquitination of KLF-1 plays an essential role in DR-induced longevity in *C. elegans* (13–15).

However, whether WWP1 contributes to DR-mediated longevity in mammals remains unclear. Therefore, in this study, we evaluated the role of WWP1 in CR-mediated longevity and metabolic adaptation using systemic *Wwp1* knockout (KO) mice.

## 2 Methods

### 2.1 Animals and diet

All animal experiments and protocols were conducted in accordance with the Fundamental Guidelines for Proper Conduct of Animal Experiments and Related Activities in Academic Research Institutions under the jurisdiction of the Ministry of Education, Culture, Sports, Science, and Technology of Japan and were approved by the Ethics Review Committee for Animal Experimentation at Tokyo University of Science (approval numbers: Y20043, Y21043, and Y22037). Mice with systemic KO of *Wwp1* (*Wwp1*^−/−^ mice) and wild-type (WT) *Wwp1*^+/+^ mice were generated as previously described (16). Genotyping was performed by PCR using KOD FX neo (Toyobo, Osaka, Japan) with the following primers: forward, 5’-AGA GGC AAG AGA ATG GCG TCA AG-3’; reverse, 5’-GGA GGT GAA AGG GTT GGA AGA ATA C-3’. Mice were maintained under specific pathogen-free conditions at 23 °C, under a 12-h light/dark cycle in the animal facility at the Faculty of Pharmaceutical Sciences, Tokyo University of Science, with free access to water and a standard diet [Charles River Formula-1: 21.9% crude protein, 5.4% crude fat, and 2.9% crude fiber (Oriental Yeast, Tokyo, Japan)]. At 10 weeks of age, WT and *Wwp1* KO mice were divided into two groups: one fed *ad libitum* (AL) and the other subjected to CR (70% of AL food intake, calculated independently for each genotype). At 24 months of age, group-housed mice were euthanized with isoflurane inhalation (Wako, Osaka, Japan), and tissues including subcutaneous WAT (sWAT), epididymal WAT (eWAT), retroperitoneal WAT, brown adipose tissue, kidney, liver, heart, quadriceps femoris muscle (QFM), extensor digitorum longus (EDL), and soleus (SOL) were collected.

### 2.2 Survival analysis

Survival curves were estimated using the Kaplan–Meier method. Within-genotype comparisons between AL and CR groups were performed using the log-rank test. A genotype-stratified log-rank test was performed using R (version 4.4.1; R Foundation for Statistical Computing, Vienna, Austria; www.r-project.org) to evaluate the overall effect of CR while accounting for genotype. Cox proportional hazards models were used to analyze the effects of genotype, diet, and their interaction on survival, and an additive Cox model including genotype and diet was used to estimate the genotype-adjusted effect of CR. Cox proportional hazards analyses were performed using GraphPad Prism (versions 10.6.1; GraphPad Software, San Diego, CA, United States).^1^

### 2.3 Respiratory quotient and energy expenditure

Open-circuit indirect calorimetry was performed using a small-animal O₂/CO₂ metabolic measurement system (MK-5000RQ; Muromachi Kikai Co., Ltd., Tokyo, Japan). Mice were individually housed in metabolic chambers maintained at a constant temperature (22–24 °C) under a 12-h light/12-h dark cycle. Animals were acclimated to the metabolic cages for 48 h prior to data collection to minimize stress-related artifacts. Oxygen consumption (VO₂) and carbon dioxide production (VCO₂) were continuously measured at 3-min intervals. The RQ was calculated as the ratio of VCO₂ to VO₂ (RQ = VCO₂/VO₂). EE was calculated using the following equation: EE = 1.44 × VO₂ × (3.815 + 1.232 × RQ).

### 2.4 Computed tomography scan

CT was performed using a third-generation CT scanner (LaTheta LCT-200; Hitachi-Aloka, Tokyo, Japan). The tube voltage was set to 50 kV, and the current was maintained at 0.5 mA. Animals were scanned in a 48 mm-wide specimen holder with a resolution of 96 μm per pixel. For all scans, a constant number of views (436), corresponding to the number of data points collected during a single 360° rotation, was used. In accordance with the manufacturer’s recommendations, radiation exposure was maintained below 40 mSv. In pilot experiments, optimal scanning conditions were determined for each tissue; the final settings were a density range of −550 to −140 Hounsfield units, a slice thickness of 192 μm, and a slice pitch of 600 μm. Visceral and subcutaneous WAT were distinguished based on their anatomical location, defined as inside (visceral) or outside (subcutaneous) the body wall muscles, including the abdominal and thoracic wall muscles.

### 2.5 Quantitative real-time PCR

Total RNA was extracted using ISOGEN II (Nippon gene, Toyama, Japan) and reverse transcription was performed using ReverTra Ace^®^ qPCR RT Master Mix (Toyobo, Osaka, Japan). Quantitative real-time PCR was performed using the CFX Connect™ Real-Time System (Bio-Rad, Hercules, CA, United States) and Thunderbird SYBR qPCR mix (Toyobo), according to manufacturer protocols. Sequences of primers used for PCR were as follows: *Wwp1* (forward, 5’-TCA GGG TGG GAA CAG AGA AAA G-3’, reverse, 5’-GCA ATT GAT TCC GCT GAG AC-3‘), Srebp-1c (forward, 5’-GGA GCC ATG GAT TGC ACA TT-3’, reverse, 5’-GGC CCG GGA AGT CAC TGT-3’), Acc (forward, 5’-TGG ATG AAC CAT CTC CGT TG-3’, reverse, 5’-CAT GTG AAA GGC CAA ACC ATC-3’), *Fasn* (forward, 5’-AGC AGG CAC ACA CAA TGG AC-3’, reverse, 5’-GAA GAA GAA AGA GAG CCG GTT G-3’), Hsl (forward, 5’-CTC CTC ATG GCT CAA CTC CTT CC-3’, reverse, 5’-AGG GGT TCT TGA CTA TGG GTG-3’), Pnpla2 (Atgl) (forward, 5’-AAG ACC CTG CCT GCT GAT TG-3’, reverse, 5’- AAA GTG GCA AGT TGT CTG AAA TGC-3’) and *Rps18* (forward, 5’-TGC GAG TAC TCA ACA CCA ACA T-3’, reverse, 5’-CTT TCC TCA ACA CCA CAT GAG C-3’). Rps18 was used as the housekeeping gene.

### 2.6 Western blotting

Cell lysates were prepared, and immunoblotting was performed as previously described (17). Briefly, tissues were lysed with sodium dodecyl sulfate (SDS) sample buffer, homogenized, boiled for 5 min, and sonicated. Lysates (15 μg protein/well) were subjected to SDS–polyacrylamide gel electrophoresis, and separated proteins were transferred onto nitrocellulose membranes. Membranes were blocked with blocking solution [2.5% skim milk, 0.25% bovine serum albumin in Tris-buffered saline with Tween 20 (25 mM Tris–HCl, pH 7.4, 140 mM NaCl, 2.5 mM KCl, and 0.1% Tween 20)] for 1 h at room temperature and then incubated with appropriate primary antibodies overnight at 4 °C. Anti-HSL (#4017), and anti-phospho-HSL (S660, #4126) antibodies were purchased from Cell Signaling Technology (Danvers, MA, USA). Anti-lamin B1 (#PM064) was purchased from MBL (Aichi, Japan). Membranes were then incubated with appropriate secondary antibodies (horseradish-peroxidase-conjugated F[ab’]2 fragment of goat anti-mouse or anti-rabbit immunoglobulin G; Jackson ImmunoResearch, West Grove, PA, United States) for 1 h at room temperature. Antibody-bound proteins were visualized using ImmunoStar LD reagent (Wako) and either an LAS3000 Image Analyzer (Fujifilm, Tokyo, Japan). Data were analyzed using MULTIGAGE software (v3.2; GE Healthcare, Chicago, WI, United States). The phosphorylation level of HSL was quantified as the ratio of phosphorylated HSL (Ser660) to total HSL. Lamin B1 was used as a loading control for representative immunoblot images.

### 2.7 Statistical analysis

One-way analysis of variance (ANOVA) followed by Tukey’s test was used to assess differences among the four groups. Statistical analyses were performed using GraphPad Prism (versions 10.6.1; GraphPad Software, San Diego, CA, United States).^2^

## 3 Results

### 3.1 *Wwp1* is not required for CR-mediated longevity in mice

**Figure 1:**
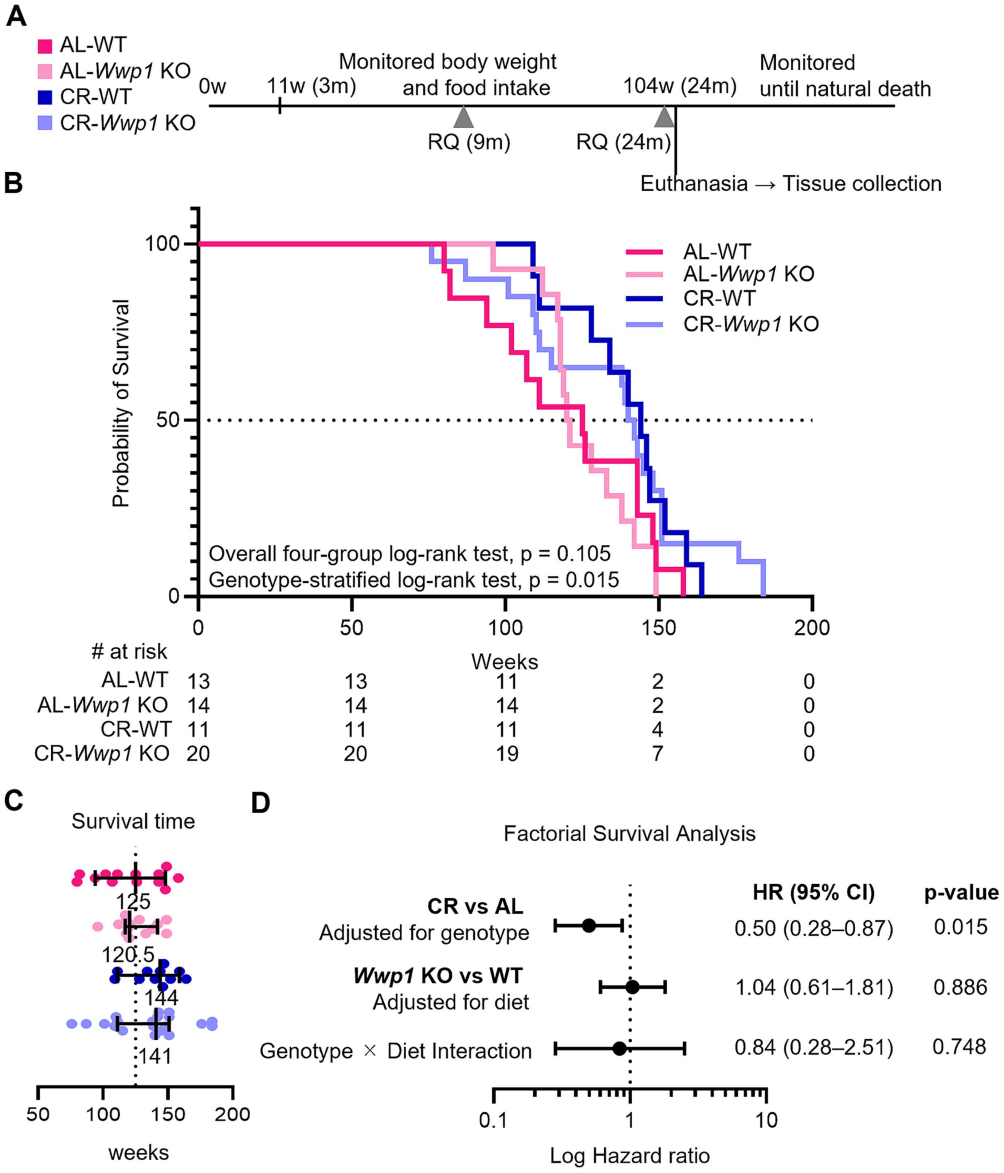
Effects of WWP1 deficiency on survival under caloric restriction. **(A)** Study design. From 11 weeks of age, mice of each genotype [wild type (WT) and *Wwp1* knockout (KO)] were assigned to *ad libitum* (AL) feeding or caloric restriction (CR; 70% of AL food intake, calculated independently for each genotype). **(B,C)** Kaplan–Meier survival curves showing overall mortality for AL-WT (*n* = 13) and CR-WT (*n* = 11) mice, and AL-*Wwp1* KO (*n* = 14) and CR-*Wwp1* KO (*n* = 20) mice, respectively. Dashed lines indicate 50% mortality. **(C)** Individual mouse lifespans (points) and mean survival time with 95% confidence intervals (CI) (error bars) for each group. Numbers indicate the median survival time (weeks) of each group. **(D)** Forest plot showing hazard ratios (HRs) with 95% CI derived from Cox proportional hazards models based on the 2 × 2 factorial design. *p*-values were determined using Cox proportional hazards regression analysis.

We examined the effects of WWP1 on the survival response to CR in male WT and *Wwp1* KO mice using a 2 × 2 factorial design with genotype (WT or *Wwp1* KO) and diet (AL or CR) as factors. Mice were randomly assigned to AL feeding or CR (70% of AL intake) beginning at 11 weeks of age and were monitored for the remainder of their natural lifespan (Figure 1A). Kaplan–Meier survival curves for all four groups are shown in Figure 1B. The overall four-group Mantel–Cox log-rank test did not reach statistical significance (*p* = 0.105). Because the study followed a 2 × 2 factorial design, we also performed a genotype-stratified log-rank test to evaluate the overall effect of CR while accounting for genotype, which showed a significant effect of CR on survival (*p* = 0.015) (Figure 1B). Median survival time was 125 weeks in AL-WT mice, 144 weeks in CR-WT mice, 120.5 weeks in AL-*Wwp1* KO mice, and 141 weeks in CR-*Wwp1* KO mice (Figure 1C). We further analyzed survival using Cox proportional hazards models based on the 2 × 2 factorial design. In the additive Cox model including genotype and diet, CR was significantly associated with reduced mortality after adjustment for genotype (HR = 0.50, 95% CI: 0.28–0.87, p = 0.015), whereas *Wwp*1 genotype itself was not associated with survival (HR = 1.04, 95% CI: 0.61–1.81, *p* = 0.886) (Figure 1D). A Cox model including genotype, diet, and their interaction showed no significant genotype-by-diet interaction (interaction HR = 0.84, 95% CI: 0.28–2.51, *p* = 0.748), indicating no statistical evidence that *Wwp1* deficiency modified the CR-associated survival response (Figure 1D). Taken together, these analyses suggest that CR was associated with improved survival after adjustment for genotype, while *Wwp1* deficiency did not significantly modify the CR-associated survival response under the present experimental conditions.

### 3.2 *Wwp1* deficiency modestly alters energy expenditure and adiposity under CR

**Figure 2:**
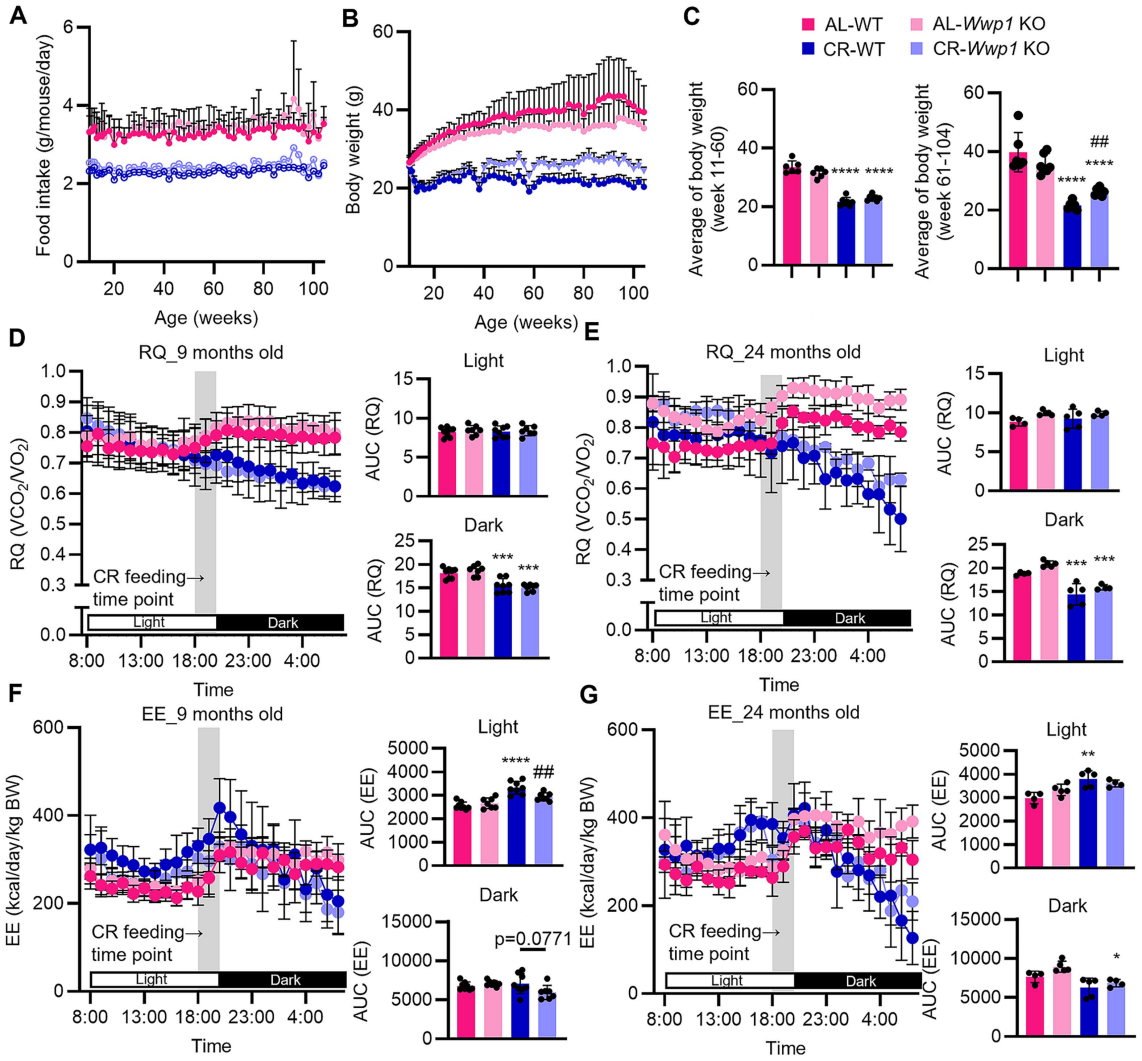
**(A)** Food intake in AL-WT (*n* = 7) and AL-*Wwp1* KO (*n* = 11) mice. Open circles in CR groups indicate values calculated as 70% of the mean food intake of the corresponding AL group. **(B-C)** Body weight over time. **(B)** Weekly body weight for AL-WT (*n* = 6), AL-Wwp1 KO (*n* = 6), CR-WT (*n* = 7), and CR-*Wwp1* KO (*n* = 7). **(C)** Mean body weight during 11–60 weeks (left) and 60–104 weeks (right). **(D-E)** Respiratory quotient (RQ; VCO_2_/VO_2_) measured over a 24-h period under 12-h light/dark cycles in 9-month-old **(D)** and 24-month-old **(E)** mice (left panels). Right panels show the area under the curve (AUC) for RQ. **(F and G)** Energy expenditure (EE: 1.44 × VO_2_ × (3.815 + 1.232 × RQ) was determined over a 24 h period with 12 h light/dark cycles in 9-month-old **(F)** and 24-month-old **(G)** mice (left panels). The right panels show the area under the curve (AUC) for EE. Values are presented as mean ± SD. Each dot represents one mouse. Differences were analyzed by one-way ANOVA with Tukey’s post hoc test. *p < 0.05, **p < 0.01, ***p < 0.001,****p < 0.0001 vs. AL; ##*p* < 0.01 vs. WT.

**Figure 3:**
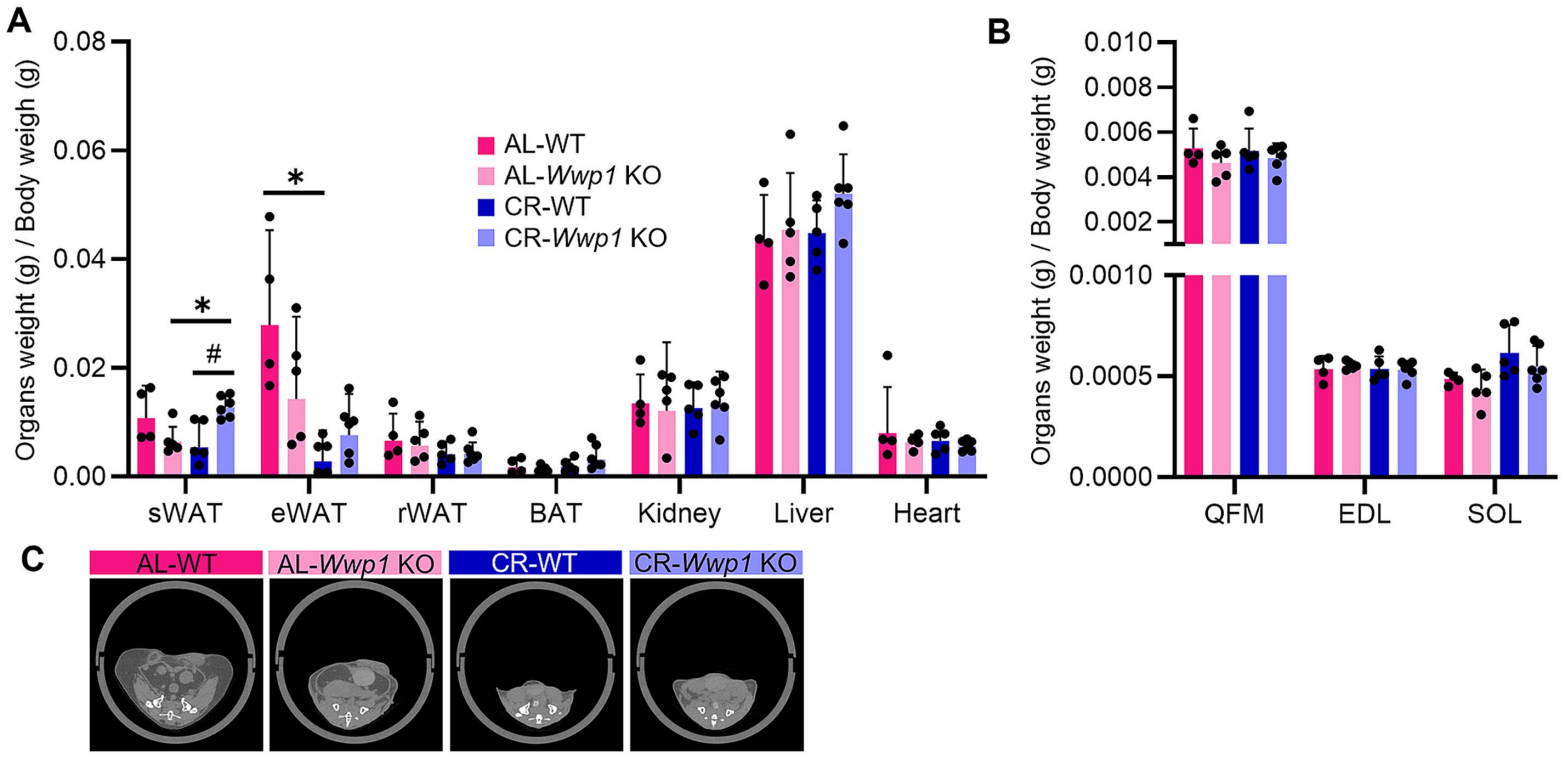
Depletion of *Wwp1* increases white adipose tissue mass during CR. **(A,B)** Organ weight normalized to body weight of subcutaneous white adipose tissue (sWAT), epididymal WAT (eWAT), retroperitoneal WAT (rWAT), brown adipose tissue (BAT), kidney, liver, heart **(A)**, and quadriceps famous muscle (QFM), extensor digitorum longus (EDL), and soleus (SOL) **(B)** in 24-month-old AL-WT (*n* = 4), AL-*Wwp1* KO (*n* = 5), CR-WT (*n* = 5), and CR-*Wwp1* KO (*n* = 6) mice. **(C)** Computed tomography (CT) axial images of eWAT and sWAT from 24-month-old mice. Values are presented as mean ± SD. Each dot represents one mouse. Differences were analyzed by one-way ANOVA with Tukey’s post hoc test. *p < 0.05 vs. AL; #*p* < 0.05 vs. WT.

Although food intake did not differ between groups, CR *Wwp1* KO mice were slightly heavier than CR WT mice, particularly during the late phase of CR (Figures 2A–C). We also measured respiratory quotient (RQ) and energy expenditure (EE) in both 9- and 24-month-old mice. RQ analyses indicated that *Wwp1* deficiency did not markedly alter the CR-associated metabolic shift in either 9- and 24-month-old mice in any group during either the light or dark cycle (Figures 2D,E; Supplementary Figure 1). On the other hand, EE analysis normalized to body weight indicated that *Wwp1* deficiency tended to decrease energy expenditure during CR especially in 9-month-old mice (Figure 2F). The difference was not observed in 24-month-old mice (Figure 2G). Next, we evaluated the effects of CR on body composition at 104 weeks of age (approximately 24 months). Under CR, sWAT, eWAT, the liver, and EDL weights were higher in *Wwp1* KO mice than in CR WT mice (Supplementary Figures 1A,B). Following normalization to body weight, the differences in both liver and EDL were no longer observed, whereas the differences in WAT remained significant (Figures 3A,B). Consistently, CT analysis showed that the CR-induced reduction in sWAT area was increased in CR *Wwp1* KO mice (Figure 3C).

### 3.3 *Wwp1* deficiency increases *de novo* fatty acid synthesis in WAT under CR

**Figure 4:**
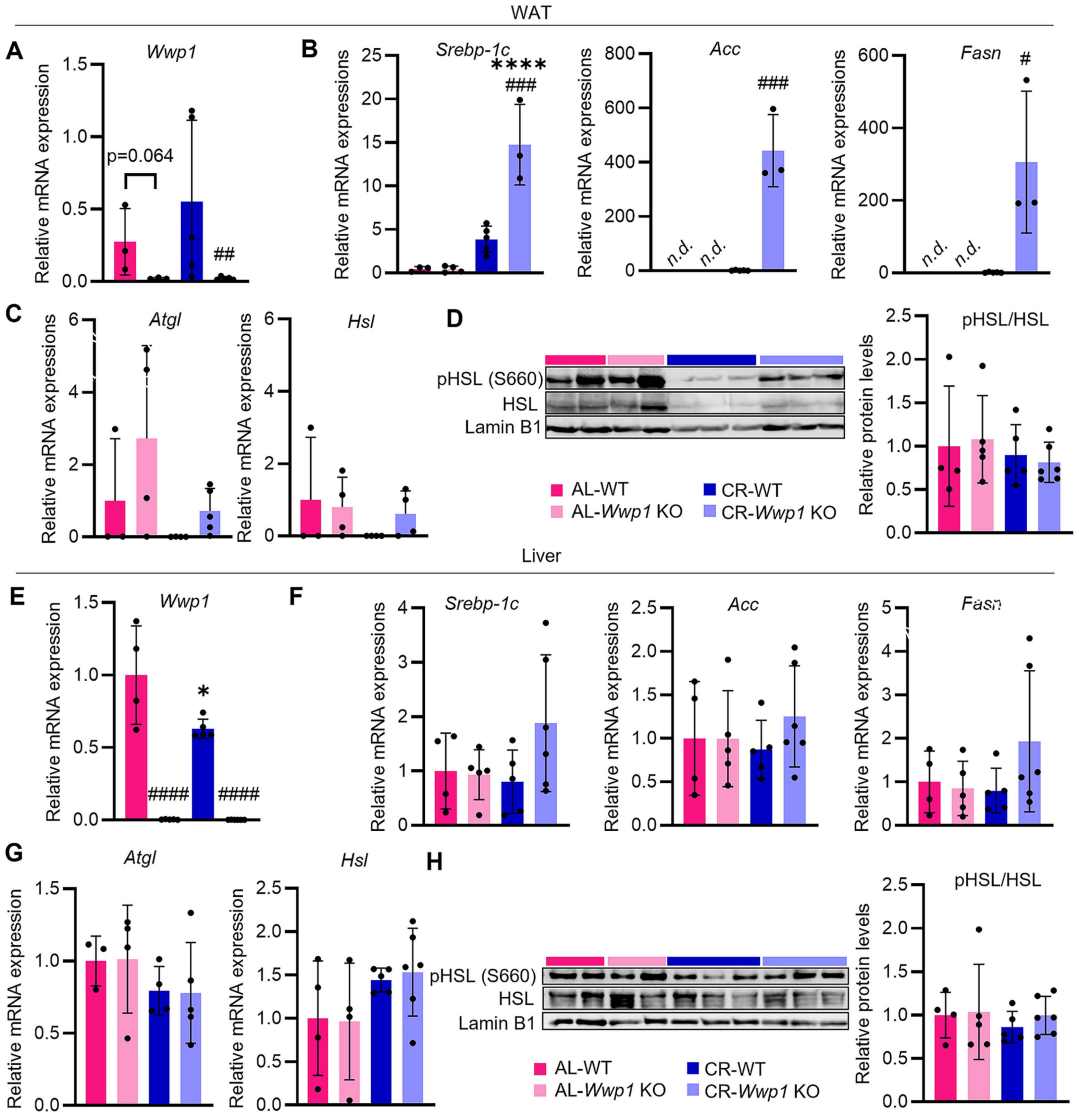
Depletion of *Wwp1* enhances CR-induced expression of fatty acid biosynthesis-related factors in WAT. Effects on white adipose tissue (WAT) **(A–D)** and liver **(E–H)** from 24-month-old AL-WT, AL-*Wwp1* KO, CR-WT, and CR-*Wwp1* KO mice. **(A–C)** mRNA expression levels of *Wwp1* **(A)**, *Srebp-1c*, *Acc*, and *Fasn* **(B)**, and Acly and Hsl **(C)** in WAT. **(D)** Protein expression ratio of phosphorylated hormone-sensitive lipase (HSL) (S660) to total HSL in WAT, determined by immunoblotting. **(E–H)** mRNA expression levels of *Wwp1* **(E)**, *Srebp-1c*, *Acc*, and *Fasn* **(F)**, and Acly and Hsl **(G)** in the liver. **(H)** Protein expression ratio of phosphorylated HSL (S660) to total HSL in WAT, determined by immunoblotting. *Rps18* was used as an internal control for real-time PCR analysis, and Lamin B1 was used as a loading control for immunoblotting. Each dot represents one mouse. Values are presented as mean ± SD. Differences were analyzed using one-way ANOVA with Tukey’s post hoc test and Student’s t-test for *Acc* and *Fasn* in WAT. *p < 0.05, ****p < 0.0001 vs. AL; ##*p* < 0.01, ###*p* < 0.001, ####*p* < 0.0001 vs. WT.

Sterol regulatory element-binding protein-1c (SREBP-1c), encoded by the *Srebf1* gene, is a master regulator of FA biosynthesis and regulates FA biosynthesis-related genes, including acetyl-CoA carboxylase (*Acc*) and fatty acid synthase (*Fasn*), by binding to SRE-1 elements (18). We previously demonstrated that the CR-associated upregulation of genes involved in FA biosynthesis within WAT may be regulated in an Srebp-1c-dependent manner, independent of GH/IGF-1 signaling (19–21). To investigate the role of WWP1, we evaluated the expression of Srebp-1c and its downstream targets in WAT from *Wwp1* KO mice in AL or CR conditions. CR-induced upregulation of *Srebp-1c*, *Acc*, and *Fasn* mRNA levels was significantly enhanced in WAT of *Wwp1* KO mice (Figures 4A,B), which may partly reflect the increased absolute mass of WAT in *Wwp1* KO mice (Figure 3A; Supplementary Figure 2). We also evaluated lipolysis by measuring the gene expression levels of adipose triglyceride lipase (Atgl) and hormone-sensitive lipase (Hsl) and its ratio of phosphorylated hormone-sensitive lipase (pHSL) to total HSL, which remained significantly unchanged in *Wwp1* KO mice (Figures 4C,D). In the liver, FA biosynthesis- and lipolysis-related gene expression and the pHSL/HSL ratio were similarly unaffected by *Wwp1* deficiency (Figures 4E–H).

## 4 Discussion

Although previous studies in a *C. elegans* model showed that reduction of *Wwp1* completely suppressed DR-induced lifespan extension and that a catalytically inactive mutant of WWP1 suppressed DR-induced longevity (13, 14), our systemic *Wwp1* KO mouse model did not show evidence that *Wwp1* deficiency significantly modified the CR-associated survival response (Figures 1B,D). In *C. elegans*, WWP-1 is orthologous to the mammalian WWP1, WWP2, and ITCH proteins. Because our mouse model lacks only WWP1 (16), the functions of WWP2 and ITCH remain intact, which may account for the discrepancy between the phenotypes observed in mice and those reported in *C. elegans*. In mammals, the effects of CR are mediated via multiple coordinated mechanisms, highlighting that the lifespan-prolonging effects are not attributable to a single molecule, but instead arise from the combined contribution of multiple factors (22). The differences in complexity between nematodes and mammals may partly account for the differing outcomes observed between the mouse model in this study and the *C. elegans* models used in previous studies. In contrast, Carrano and colleagues reported that the intestine is a major site in which WWP1 regulates CR-induced longevity through ubiquitination of KLF-1 in *C. elegans* (14). As this study used systemic *Wwp1* KO mice, future research using tissue-specific *Wwp1* KO models is warranted to evaluate CR-induced longevity.

Previous studies have shown that CR induces the expression of Srebp-1c-regulated genes, particularly those involved in FA biosynthesis, and that Srebp-1c mediates the effects of CR in WAT (19–21, 23). Increased Srebp-1c enhances FA biosynthesis and mitochondrial biogenesis in a PPARγ coactivator-1α (PGC-1α)–dependent manner by directly binding sterol regulatory elements in target gene promoters (21). Such metabolic remodeling of WAT is thought to partially contribute to the pro-longevity effects of CR. In this study, we found that *Wwp1* deficiency increased CR-induced expression of *Srebp-1c* and genes involved in *de novo* FA biosynthesis in WAT of 24-month-old mice (Figure 4B). Consistent with these findings, CR-*Wwp1* KO mice exhibited increased WAT mass in 24 months of age (Figure 3A; Supplementary Figure 2). Together with the tendency toward reduced energy expenditure in *Wwp1* KO at 9 months of age (Figure 2F), these findings suggest that WWP1 initially suppresses systemic energy expenditure at younger ages during CR and subsequently suppresses lipid storage and retention in WAT at older ages during CR. Such metabolic adaptations may contribute to the increased body weight observed in CR-*Wwp1* KO mice independently of food intake (Figures 3A–C).

Collectively, our results demonstrate that, unlike in *C. elegans*, WWP1 is not required for CR-induced longevity in mammals; rather, it acts as a partial suppressor of CR-driven *de novo* FA biosynthesis, energy storage, and lipid retention in WAT at least after middle age during CR. Further time-course analyses from younger ages will be necessary to better understand the effects of *Wwp1* deficiency during. This study highlights species-specific differences in CR regulatory mechanisms and reveals a previously unrecognized role of WWP1 in WAT metabolic adaptation under CR.

## Data Availability

The raw data supporting the conclusions of this article will be made available by the corresponding author upon request.

## Footnotes

1
www.graphpad.com

2
www.graphpad.com

## Supplementary material

Supplementary Figure 1 Effect of Wwp1 deficiency on CR-associated metabolic shift in an age-dependent manner. **(A,B)** Average respiratory quotient (RQ; VCOâ‚/VOâ‚) measured over a 24-h period under 12-h light/dark cycles in 9- and 24-month-old mice during the light **(A)** and dark **(B)** cycles. Each dot represents one mouse. Values are presented as mean Â ± SD.

Supplementary Figure 2 Effect of Wwp1 deficiency on organ weight. **(A,B)** Organ weight of subcutaneous white adipose tissue (sWAT), epididymal WAT (eWAT), retroperitoneal WAT (rWAT), brown adipose tissue (BAT), kidney, liver, heart **(A)**, and quadriceps famous muscle (QFM), extensor digitorum longus (EDL), and soleus (SOL) **(B)** in 24-month-old AL-WT (*n* = 4), AL-Wwp1 KO (*n* = 5), CR-WT (*n* = 5), and CR-Wwp1 KO (*n* = 6) mice.
