# Supplementary figures and images for: WWP1 modulates metabolic adaptation in white adipose tissue but does not significantly modify caloric restriction-induced longevity in mice
Source: Front Nutr. 2026 Jun 30;13:1846488. doi: 10.3389/fnut.2026.1846488 (PMC13364857; doi:10.3389/fnut.2026.1846488)

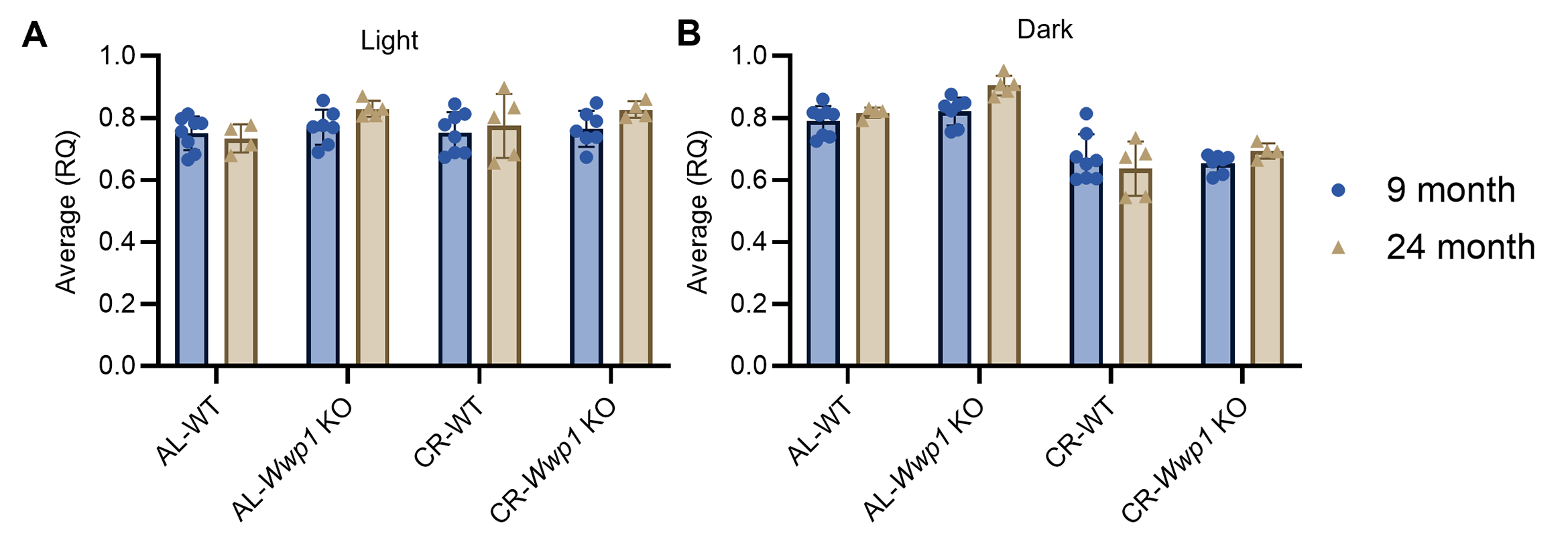

Supplement: Supplementary Figure 1 — Effect of Wwp1 deficiency on CR-associated metabolic shift in an age-dependent manner. (A,B) Average respiratory quotient (RQ; VCOâ‚/VOâ‚) measured over a 24-h period under 12-h light/dark cycles in 9- and 24-month-old mice during the light (A) and dark (B) cycles. Each dot represents one mouse. Values are presented as mean Â ± SD. [file Image_1.TIF]

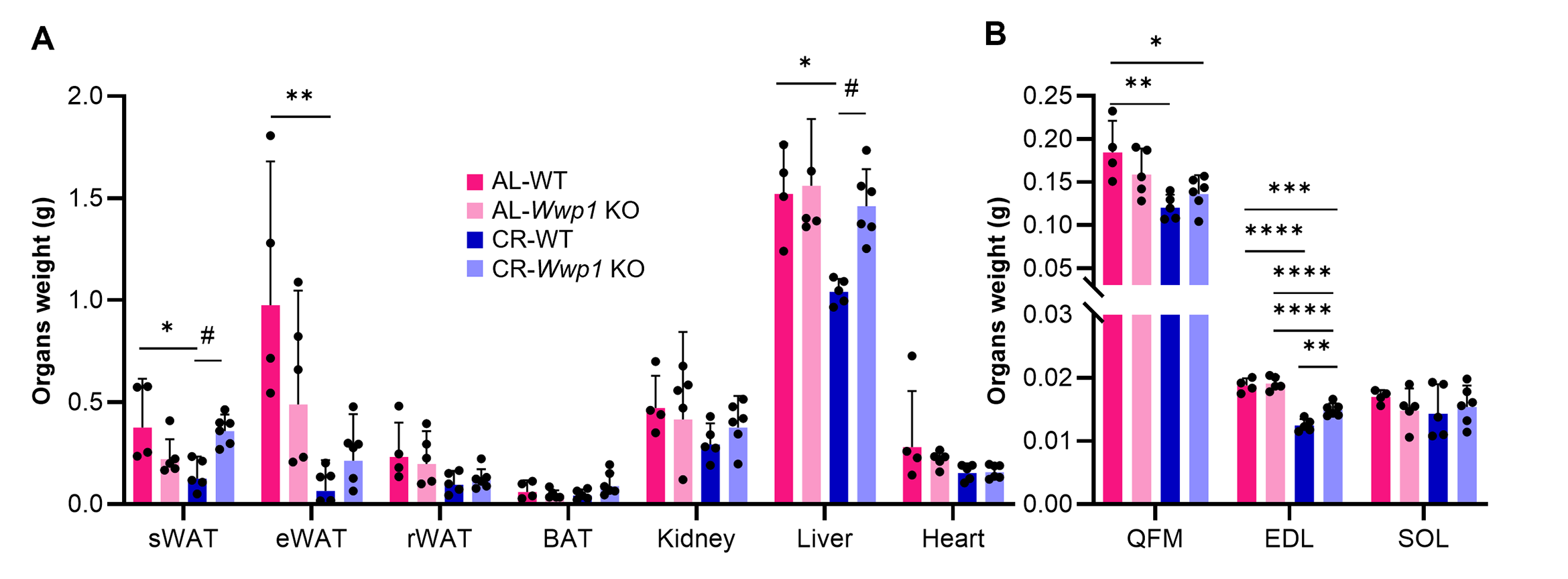

Supplement: Supplementary Figure 2 — Effect of Wwp1 deficiency on organ weight. (A,B) Organ weight of subcutaneous white adipose tissue (sWAT), epididymal WAT (eWAT), retroperitoneal WAT (rWAT), brown adipose tissue (BAT), kidney, liver, heart (A), and quadriceps famous muscle (QFM), extensor digitorum longus (EDL), and soleus (SOL) (B) in 24-month-old AL-WT (n = 4), AL-Wwp1 KO (n = 5), CR-WT (n = 5), and CR-Wwp1 KO (n = 6) mice. [file Image_2.TIF]
